# Use of health services and medicines amongst Australian war veterans: a comparison of young elderly, near centenarians and centenarians

**DOI:** 10.1186/1471-2318-10-83

**Published:** 2010-11-04

**Authors:** Elizabeth E Roughead, Lisa M Kalisch, Emmae N Ramsay, Philip Ryan, Andrew L Gilbert

**Affiliations:** 1 Quality Use of Medicines and Pharmacy Research Centre, Sansom Institute, School of Pharmacy and Medical Sciences, University of South Australia, Adelaide, Australia; 2 Data Management and Analysis Centre, Discipline of Public Health, The University of Adelaide, Adelaide, Australia

## Abstract

**Background:**

Age and life expectancy of residents in many developed countries, including Australia, is increasing. Health resource and medicine use in the very old is not well studied. The purpose of this study was to identify annual use of health services and medicines by very old Australian veterans; those aged 95 to 99 years (near centenarians) and those aged 100 years and over (centenarians).

**Methods:**

The study population included veterans eligible for all health services subsidised by the Department of Veterans' Affairs (DVA) aged 95 years and over at August 1^st ^2006. A cohort of veterans aged 65 to 74 years was identified for comparison. Data were sourced from DVA claims databases. We identified all claims between August 1^st ^2006 and July 31^st ^2007 for medical consultations, pathology, diagnostic imaging and allied health services, hospital admissions, number of prescriptions and unique medicines. Chi squared tests were used to compare the proportion of centenarians (those aged 100 years and over) and near centenarians (those aged 95 to 99 years) who accessed medicines and health services with the 65 to 74 year age group. For those who accessed health services during follow up, Poisson regression was used to compare differences in the number of times centenarians and near centenarians accessed each health service compared to 65 to 74 year olds.

**Results:**

A similar proportion (98%) of centenarians and near centenarians compared to those aged 65 to 74 consulted a GP and received prescription medicine during follow up. A lower proportion of centenarians and near centenarians had claims for specialist visits (36% and 57% respectively), hospitalisation (19% and 24%), dental (12% and 18%), physiotherapy (13% and 15%), pathology(68% and 78%) and diagnostic imaging services (51% and 68%) (p < 0.0001) and a higher proportion had claims for care plans (19% and 25%), occupational therapy (15% and 17%) and podiatry services (54% and 58%) (p < 0.0001). Compared to those aged 65 to 74, a lower proportion of centenarians and near centenarians received antihypertensives, lipid lowering therapy, antiinflammatories, and antidepressants (p < 0.0001) and a higher proportion received antibiotics, analgesics, diuretics, laxatives, and anti-anaemics (p < 0.0001).

**Conclusions:**

Medical consultations and medicines are the health services most frequently accessed by Australian veteran centenarians and near centenarians. For most health services, the proportion of very old people who access them is similar to or less than younger elderly. Our results support the findings of other studies which suggest that longevity is not necessarily associated with excessive health service use.

## Background

The average age and life expectancy of the Australian population is increasing. In the 1970s life expectancy for males was around 68 years and 75 years for females; by the 2000s this had increased to 78 years for men and 83 years for women [1]. The proportion of the Australian population aged over 65 years increased from 11% in 1988 to 13% in 2008 [2]. This is largely due to a rise in the number of people aged 85 years and over. Over the 20 years between June 1988 and June 2008 the proportion of the Australian population aged 85 years and over rose by 165%; while the overall size of the Australian population increased by only 29% during this time [2]. The number of people aged 95 to 99 years (near centenarians), and the number of centenarians (those aged 100 years and over) nearly tripled in this 20 year period [2]. The health of Australian centenarians and near centenarians has not been widely studied.

International studies have reported the mean number of comorbidities amongst centenarians ranges from two to four [3-6]. Comorbidities associated with mortality and morbidity amongst the elderly, including heart disease, stroke and diabetes, are less prevalent amongst centenarians and tend to be diagnosed after age 80 amongst centenarians who suffer them [5-8].

The proportion of centenarians using prescription medicines ranges from 54% to 95% [3,9,10]. Types of medicines commonly used by this age group are drugs for hypertension and cardiovascular and gastrointestinal conditions [3,9-11]. Health resource use by centenarians and near centenarians is not widely reported. A study of Greek centenarians found that 79% had visited their general practitioner (GP) in the past year[12] and a study of Danish centenarians found that 42% contacted their GP in the previous three months [3]. Nearly all centenarians have been hospitalised at least once in their lifetime [3,12]. A 2003 analysis by age group of health service use by Australian veterans found that amongst the 95 years and older age group 94% saw their GP during the year of follow-up [13]. A smaller proportion of this age group (62%) saw specialists, and just over half (52%) aged 95 years and over were hospitalised [13]. Medicine use by those aged 95 and over was common, with 89% having prescription medicines dispensed [13]. However, centenarians and near centenarians as two distinct age groups were not studied.

Australians have access to free treatment at public hospitals and free or subsidised treatment by GPs and some allied health professionals under Medicare, funded by the Australian government [14]. Access to medicines is subsidised under the Pharmaceutical Benefits Scheme (PBS) and Repatriation Pharmaceutical Benefits Scheme (RPBS) [14]. Patients pay a co-payment for medicines with the remainder of the cost met by the Australian government [14]. Older people tend to use more medicines than younger people, and the contribution of an ageing population to increased government expenditure on medicines subsidy has been acknowledged [15]. The potential for the ageing population to lead to an increase in demand for Medicare funded health services, and the associated increase in government funding costs has also been discussed [16,17]. However, the influence of the rapidly growing number of centenarians and near centenarians on increased medicine and health resource use in Australia has had limited study and the available studies have not considered centenarians as a distinct age group. This is an important area for research, because population projections suggest that centenarians will number 13,000 by 2020,[18] compared to 6,000 in 2010. In this study, we aimed to identify the annual use of health services and medicines by Australian veteran centenarians (those aged 100 years and over) and near centenarians (those aged 95 to 99 years).

## Methods

Data for this study were sourced from the Department of Veterans' Affairs (DVA) health claims databases. The DVA treatment population is comprised mainly of Australian defence force veterans and their eligible dependants, including spouses, widows or widowers, and children. Over two thirds of the DVA treatment population have served in the Australian defence force, with 89% of those who served being male [19]. Reflecting this, in 2009 59% of the overall DVA treatment population were male and over 70% were aged 70 years or over [20]. Eligible veterans who hold a DVA gold card receive all health services and medicines subsidised by DVA [19]. White card holders receive DVA subsidy for health services and medicines for the treatment of specific service related conditions only [19]. The DVA claims databases contain details of all prescription medicines, medical and allied health services and hospitalisations provided to veterans for which DVA pay a subsidy. The data file contains 80 million pharmacy records, 200 million medical and allied health service records and over 6 million hospital records for a treatment population of 310,000 veterans. DVA maintain a client file, which includes data on gender, date of birth and date of death.

Subjects for this study were DVA gold card holders, eligible for all DVA funded health services, who were 96 years of age or older on August 1^st ^2007. We retrospectively extracted data on all claims in the year prior (August 1^st ^2006 to July 31^st ^2007) for the following health services: general practice consultations, specialist consultations, care planning services (including GP care plans, discharge plans and multidisciplinary case conferences), pathology services, diagnostic imaging services, allied health services (including occupational therapist, speech therapist, dietician, physiotherapy, social work and podiatrist services), dental consultations, psychologist services and hospital admissions. In addition, we extracted counts of the number of prescriptions and unique medicines (defined by ATC code) dispensed. The proportion of subjects accessing each health service and the proportion of subjects dispensed each class of medicine, defined by the third digit ATC code, was assessed. Amongst those who accessed health services, the mean number of times each service was accessed was determined.

Sub-group analysis was undertaken by age group: near centenarians (those aged 95 to 99 years) and centenarians (those aged 100 years and over). This age group definition allowed focus on Australian centenarians, an age group for which little prior research has been conducted [21]. The influence of the increasing number of centenarians and near centenarians medicine and health resource use in Australia has had limited study. Therefore, to enable comparison of health service use by centenarians and near centenarians with health service use by younger elderly people, a comparison cohort of DVA gold card holders aged 65 to 74 years was identified in an identical manner. This age group was chosen because more than half of all Australians aged 65 years and over are aged between 65 and 74 years.

Chi squared tests were used to compare the proportion of centenarians and near centenarians who accessed medicines and health services with the 65 to 74 year age group. For those who accessed health services during follow up, Poisson regression was used to compare differences in the mean number of times centenarians and near centenarians accessed each health service compared to 65 to 74 year olds. No covariates were included in the Poisson regression. Post hoc pair wise comparisons were undertaken to compare the three age groups, with no adjustments made for multiple comparisons. All analyses were undertaken using SAS, V9.1 (SAS institute, Cary, North Carolina, USA). Ethics approval for the study was obtained from the Department of Veterans' Affairs Human Research Ethics Committee and the University of South Australia Human Research Ethics Committee.

## Results

The study population consisted of 1,734 veterans aged 95 years and over. The majority (n = 1,624, 93.6% of the very old cohort) were aged between 95 and 99 years (near centenarians), while 110 (6.3%) were aged 100 years or more (centenarians). Two thirds of near centenarians (n = 1,100, 67.7%) were female, while 82% of centenarians (n = 90) were female. Just under half of near centenarians (n = 800, 49%) and 67% of centenarians (n = 74) lived in residential care during the study period.

The proportion of centenarians and near centenarians who consulted a GP and had a prescription medicine dispensed during the year of follow up was similar to those aged 65 to 74 years, 98% (table 1). A lower proportion of centenarians and near centenarians consulted a specialist (36% and 57% respectively, table 1).

**Table 1:** Number of subjects with at least one claim for service between August 1^st ^2006 and July 31^st ^2007

Service type	Veterans aged 65-74 years n = 20,413	Near centenarians (95-99 years) n = 1,624	Centenarians (≥ 100 years) n = 110	Chi square*
GP visit	19,872 (97.3%)	1,590 (97.9%)	108 (98.2%)	2.1071; p = 0.35

Specialist visit	14,848 (72.7%)	917 (56.5%)	40 (36.4%)	261.2; p < 0.0001

Health care plan	583 (2.9%)	412 (25.4%)	21 (19.1%)	1795.1; p < 0.0001

GP plan	3,062 (15.0%)	205 (12.6%)	10 (9.1%)	9.6; p = 0.008

Case conference	174 (0.85%)	60 (3.7%)	4 (3.6%)	121.1; p < 0.0001

Pathology	16,985 (83.2%)	1,260 (77.6%)	75 (68.2%)	49.6; p < 0.0001

Diagnostic imaging	15,782 (77.3%)	1,097 (67.5%)	56 (50.9%)	119.8; p < 0.0001

Occupational therapy	1,686 (8.3%)	271 (16.7%)	16 (14.5%)	136.0; p < 0.0001

Speech therapy	46 (0.2%)	9 (0.6%)	0	6.8; p = 0.0327

Dietician	551 (2.7%)	17 (1.0%)	2 (1.8%)	16.6; p = 0.0002

Dental	8,697 (42.6%)	291 (17.9%)	13 (11.8%)	418.1; p < 0.0001

Physiotherapy	5,037 (24.7%)	236 (14.5%)	14 (12.7%)	92.7; p < 0.0001

Social work	27 (0.1%)	2 (0.1%)	0	0.2; p = 0.9257

Psychologist	248 (1.2%)	3 (0.2%)	0	15.5; p = 0.0004

Podiatry	7,563 (37.0%)	947 (58.3%)	59 (53.6%)	297.1; p < 0.0001

Hospital admission	6,795 (33.3%)	388 (23.9%)	21 (19.1%)	69.6; p < 0.0001

Prescription	19,778 (96.9%)	1,590 (97.9%)	108 (98.2%)	5.8; p = 0.054

*df = 2 for all comparisons

Compared to the 65 to 74 year age group, a lower proportion of centenarians had claims for hospitalisation (19% vs. 33%), dental services (12% vs. 43% ), physiotherapy services (13% vs. 25%), pathology (68%vs. 83%) and diagnostic imaging services (51%vs. 77%) during the study period. A higher proportion of centenarians compared to those aged 65 to 74 years accessed health care plans (19% vs. 3%), case conferences (4% vs. 0.9%), occupational therapy (15% vs. 8%) and podiatry services (54% vs. 37%) (table 1).

Compared to the 65 to 74 year age group, a lower proportion of near centenarians had claims for hospitalisation (24% vs. 33%), dental services (18% vs. 43%), physiotherapy services (15% vs. 25%), pathology (78% vs. 83%) and diagnostic imaging services (68% vs. 77%) during the study period. A higher proportion of near centenarians compared to the 65 to 74 year age group accessed health care plans (25% vs. 3%), case conferences (4% vs. 0.9%), occupational therapy (17% vs. 8%) and podiatry services (58% vs. 37%) (table 1).

For the proportion of near centenarians who accessed at least one service, they tended to have more GP and specialist visits, received more prescriptions and unique medicines and had more physiotherapy visits than those aged 65 to 74 who accessed these services (table 2).

**Table 2:** Mean (95% confidence interval) number of times 65-74 year olds and 95-99 year olds accessed each medical and allied health service

Service type	Veterans aged 65-74 years	Near centenarians (95-99 years)	Rate ratio (95% CI)* (Near centenarians compared to 65-74 years)
GP visit	10.80 (10.69,10.92)	14.82 (14.36,15.30)	1.37 (1.33, 1.42) p < 0.0001

Specialist visit	6.25 (6.14, 6.37)	8.31 (7.79, 8.86)	1.33 (1.24, 1.42) p < 0.0001

Health care plan	1.01 (1.00, 1.01)	1.02 (1.02, 1.03)	1.02 (1.01, 1.03) p < 0.0001

GP plan	1.85 (1.81, 1.88)	1.55 (1.43, 1.67)	0.84 (0.77, 0.91) p < 0.0001

Case conference	1.34 (1.27, 1.43)	1.20 (1.08, 1.34)	0.89 (0.79, 1.01) p = 0.0741

Pathology	15.78 (15.50,16.06)	16.37 (15.36,17.44)	1.04 (0.97, 1.11) p = 0.2746

Diagnostic imaging	7.68 (7.53, 7.83)	7.60 (7.05, 8.19)	0.99 (0.92, 1.07) p = 0.7906

Occupational therapy	2.51 (2.42, 2.61)	2.79 (2.56, 3.05)	1.11 (1.01, 1.22) p = 0.0310

Dietician	3.97 (3.69, 4.26)	3.18 (2.02, 4.99)	0.80 (0.51, 1.26) p = 0.3406

Dental	1.53 (1.52, 1.55)	1.41 (1.33, 1.49)	0.92 (0.87, 0.97) p = 0.0024

Physiotherapy	14.86 (14.43,15.29)	22.64 (20.32,25.23)	1.52 (1.36, 1.70) p < 0.0001

Podiatry	5.82 (5.76, 5.89)	5.55 (5.37, 5.74)	0.95 (0.92, 0.99) p = 0.0073

Hospital admission	2.26 (2.20, 2.32)	1.80 (1.59, 2.05)	0.80 (0.70, 0.91) p = 0.0007

Number of prescriptions	50.70 (50.21,51.20)	55.18 (53.40,57.02)	1.09 (1.05, 1.13) p < 0.0001

Number of unique medicines	11.53 (11.43,11.64)	12.65 (12.27,13.03)	1.10 (1.06, 1.13) p < 0.0001

*Calculated using Poisson regression.

For the proportion of centenarians who claimed at least one service, they had a higher number of GP and physiotherapy visits than those aged 65 to 74 who accessed these services (table 3). For all other health services studied, the number of times centenarians accessed them was no different to those aged 65 to 74 (table 3).

**Table 3:** Mean (95% confidence interval) number of times 65-74 year olds and ≥ 100 year olds accessed each medical and allied health service

Service type	Veterans aged 65-74 years	Centenarians (≥ 100 years)	Rate ratio (95% CI)* (Centenarians compared to 65-74 years)
GP visit	10.80 (10.69,10.92)	15.26 (13.54,17.19)	1.41 (1.25, 1.59) p < 0.0001

Specialist visit	6.25 (6.14, 6.37)	7.48 (5.39,10.37)	1.20 ( 0.86, 1.66) p = 0.2860

Health care plan	1.01 (1.00, 1.01)	1.00 (0.97, 1.03)	0.99 (0.97, 1.03) p = 0.7376

GP plan	1.85 (1.81, 1.88)	1.50 (1.04, 2.16)	0.81 (0.56, 1.17) p = 0.2619

Case conference	1.34 (1.27, 1.43)	1.25 (0.83, 1.89)	0.93 (0.61, 1.41) p = 0.7326

Pathology	15.78 (15.50,16.06)	11.68 (8.58,15.89)	0.74 (0.54, 1.01) p = 0.0559

Diagnostic imaging	7.68 (7.53, 7.83)	6.75 (4.75, 9.59)	0.88 (0.62, 1.25) p = 0.4735

Occupational therapy	2.51 (2.42, 2.61)	2.19 (1.45, 3.30)	0.87 (0.58, 1.31) p = 0.5073

Dietician	3.97 (3.69, 4.26)	1.00 (0.10,10.40)	0.25 (0.02, 2.63) p = 0.2492

Dental	1.53 (1.52, 1.55)	1.23 (0.93, 1.63)	0.80 (0.61, 1.06) p = 0.1249

Physiotherapy	14.86 (14.43,15.29)	24.93 (16.33,38.06)	1.68 (1.10, 2.56) p = 0.0167

Podiatry	5.82 (5.76, 5.89)	5.71 (5.01, 6.51)	0.98 (0.86, 1.12) p = 0.7729

Hospital admission	2.26 (2.20, 2.32)	1.52 (0.84, 2.75)	0.67 (0.37, 1.22) p = 0.1923

Number of prescriptions	50.70 (50.21,51.20)	47.36 (41.35,54.25)	0.93 (0.82, 1.07) p = 0.3261

Number of unique medicines	11.53 (11.43,11.64)	11.06 (9.77,12.51)	0.96 (0.85, 1.09) p = 0.5041

*Calculated using Poisson regression

Compared to those aged 65 to 74, a lower proportion of centenarians had claims for medicines acting on the renin-angiotensin system (26% vs. 51%), lipid lowering therapy (3% vs. 49%), calcium channel blockers (15% vs. 24%), beta blockers (11% vs. 23%), antiinflammatories (10% vs. 32%), psychoanaleptics (12% vs. 27%), systemic corticosteroids (5% vs. 13%), antihistamines (2% vs. 11%) and medicines for airways disease (13% vs. 25%), gout (6% vs. 11%) and diabetes (4% vs. 14%)(table 4). Compared to those aged 65 to 74, centenarians had a higher proportion with at least one claim for antibiotics (66% vs. 56%), analgesics (67% vs. 47%), psycholeptics (42% vs. 32%), eye preparations (57% vs. 30%), diuretics (56% vs. 18%), laxatives (34% vs. 11%), cardiac therapy (36% vs. 14%), mineral supplements (23% vs. 7%) and anti-anaemics (20% vs. 7%) (table 4).

**Table 4:** Number of subjects dispensed each medicine class* between August 1^st ^2006 and July 31^st ^2007

Medicine group (ATC code)	Veterans aged 65-74 years n = 20,413	Near centenarians (95-99 years) n = 1,624	Centenarians (≥ 100 years) n = 110	Chi square**
Antibacterial for systemic use (J01)	11,346 (55.6%)	1,066 (65.6%)	73 (66.4%)	66.4; p < 0.0001

Analgesics (N02)	9,642 (47.2%)	1,042 (64.2%)	74 (67.3%)	188.0; p < 0.0001

Agents acting on renin-angiotensin system (C09)	10,497 (51.4%)	670 (41.3%)	29 (26.4%)	88.1; p < 0.0001

Antithrombotic agents (B01)	7,659 (37.5%)	786 (48.4%)	42 (38.2%)	75.3; p < 0.0001

Drugs for acid related disorders (A02)	9,209 (45.1%)	732 (45.1%)	45 (40.9%)	0.78; p = 0.68

Ophthalmologicals (S01)	6,189 (30.3%)	852 (52.5%)	63 (57.3%)	370.8; p < 0.0001

Serum lipid reducing agents (C10)	10,024 (49.1%)	173 (10.7%)	3 (2.7%)	978.9; p < 0.0001

Psycholeptics (N05)	6,488 (31.8%)	704 (43.3%)	46 (41.8%)	95.7; p < 0.0001

Corticosteroids for dermatological use (D07)	5,348 (26.2%)	447 (27.5%)	26 (23.6%)	1.8; p = 0.41

Diuretics (C03)	3,627 (17.8%)	687 (42.3%)	61 (55.5%)	660.1; p < 0.0001

Calcium channel blockers (C08)	4,950 (24.2%)	334 (20.6%)	16 (14.5%)	16.6; p = 0.0003

Beta blocking agents (C07)	4,660 (22.8%)	318 (19.6%)	12 (10.9%)	17.6; p < 0.0001

Psychoanaleptics (N06)	5,475 (26.8%)	368 (22.7%)	13 (11.8%)	25.5; p < 0.0001

Cardiac therapy (C01)	2,931 (14.4%)	550 (33.9%)	39 (35.5%)	459.9; p < 0.0001

Anti-inflammatory agents (M01)	6,563 (32.2%)	223 (13.7%)	11 (10.0%)	262.2; p < 0.0001

Drugs for obstructive airways disease (R03)	5,050 (24.7%)	268 (16.5%)	14 (12.7%)	63.6; p < 0.0001

Laxatives (A06)	2,226 (10.9%)	643 (39.6%)	37 (33.6%)	1126.9;p < 0.0001

Drugs for treatment of bone diseases (M05)	1,866 (9.1%)	246 (15.1%)	9 (8.2%)	62.9; p < 0.0001

Corticosteroids for systemic use (H02)	2,653 (13.0%)	161 (9.9%)	5 (4.5%)	19.5; p < 0.0001

Mineral supplements (A12)	1,499 (7.3%)	291 (17.9%)	25 (22.7%)	254.6; p < 0.0001

Antianaemic preparations (B03)	1,507 (7.4%)	247 (15.2%)	22 (20.0%)	146.4; p < 0.0001

Otologicals (S02)	2,012 (9.9%)	244 (15.0%)	11 (10.0%)	43.7; p < 0.0001

Antigout preparations (M04)	2,175 (10.7%)	137 (8.4%)	6 (5.5%)	10.9; p = 0.004

Drugs used in diabetes (A10)	2,917 (14.3%)	72 (4.4%)	4 (3.6%)	134.3; p < 0.0001

Antihistamines for systemic use (R06)	2,268 (11.1%)	109 (6.7%)	2 (1.8%)	39.5; p < 0.0001

*Data for medicine groups dispensed to ≤10% of all veterans not shown

**df = 2 for all comparisons

Compared to those aged 65 to 74, a lower proportion of near centenarians had claims for medicines acting on the renin-angiotensin system (41% vs. 51%), lipid lowering therapy (11% vs. 49%), antiinflammatories (14% vs. 32%), antihistamines (7% vs. 11%) and medicines for airways disease (17% vs. 25%) and diabetes (4% vs. 14%) (table 4). Compared to those aged 65 to 74, near centenarians had a higher proportion with at least one claim for antibiotics (66% vs. 56%), analgesics (64% vs. 47%), antithrombotics (48% vs. 38%), cardiac therapy (34% vs. 14%), psycholeptics (43% vs. 32%), eye preparations (53% vs. 30%), diuretics (42% vs. 18%), laxatives (40% vs. 11%), mineral supplements (18% vs. 7%), medicines for bone disease (15% vs. 9%) and anti-anaemics (15% vs. 7%) (table 4).

## Discussion

Our study characterised health resource and medicine use by Australian centenarians and near centanarians. We found that, while use is frequent with 98% accessing GP services and using prescription medicines, for most health services studied the proportion of centenarians and near centenarians who accessed them was similar to or less than the proportion of veterans aged 65 to 74 who accessed them. Amongst centenarians and near centenarians who accessed one or more health service, in most cases they did so a similar number of times in comparison to younger people.

The need to know more about the health status of Australian centenarians to inform health and public policy development has been highlighted [21]. Recent studies provide evidence to support the notion that longevity is not necessarily associated with excessive health resource use and costs. An American study found that although overall health expenditure increased with age, this was largely due to increased use of nursing homes by the elderly [22]. They found that within the elderly population, the rate of acute health care use and expenditure decreased with increasing age [22]. An Australian study found similar results. Using Medicare claims data, they found that high health service usage and costs were associated with death, but not increasing age [23]. This finding has also been reflected in British[24,25] and New Zealand[26] studies. Our findings provide further evidence that suggests, in comparison to younger elderly people, Australians centenarians and near centenarians are not excessive users of health services.

Our study highlighted that the health service most commonly accessed by centenarians and near centenarians was GP visits; accessed by 98% of near centenarians and centenarians during the study period. This was slightly higher than the rate of GP visits by centenarians in other countries; with a study of Greek centenarians finding that 79% had visited their GP in the previous year [12], and a Danish study finding that 42% of centenarians had visited their GP in the previous three months [3]. Use of other health services by centenarians and near centenarians has not been extensively reported previously. A study of Danish centenarians showed the mean number of hospitalisations per person per three years at the ages 97 to 99 was 1.45[3], equivalent to 0.48 hospitalisations per person per year; while a study of centenarians in the USA found a mean of 0.6 hospital admissions per centenarian per year [4]. Our study reported results slightly differently and showed that, amongst those hospitalised, the mean number of admissions during the year was 1.5 and 1.8 amongst centenarians and near centenarians respectively. Expression of our results in a comparable way to the prior research provides an overall mean of 0.29 hospitalisations per centenarian per year and 0.43 hospitalisations per near centenarian per year; comparable to the rate of hospitalisation amongst Danish near centenarians and lower than the rate of hospitalisation amongst American centenarians.

Use of medicines is the most common health activity undertaken by Australians,[27] and our results show that centenarians and near centenarians use multiple medicines at similar levels to their younger elderly counterparts. However, the types of medicine used differ greatly. For example, 3% of centenarians received lipid lowering medicines compared to 49% of those aged 65 to 74. Calcium channel blockers and beta blockers were dispensed to 15% and 11% of centenarians, compared to 24% and 23% of those aged 65 to 74. This may reflect lower prevalence of chronic conditions in centenarians for which these medicines are used to treat, or conversely, it may reflect under-treatment of chronic conditions in centenarians. There is evidence of lower prevalence of hypertension amongst centenarians,[6,28] and studies of Italian and Japanese centenarians have demonstrated low total cholesterol in this age group [28]. Prevalence of diabetes amongst centenarians is also low in some studies [6,8]. However, other studies have suggested that there is under treatment of chronic conditions in the very old, [23,29-32] in some cases due to under diagnosis [33]. Diagnoses are not included in the DVA dataset, so we were unable to determine whether our results indicate differing prevalence of chronic diseases by age group or under-treatment in centenarians.

Centenarians and near centenarians are rarely included in randomised controlled trials, meaning that data regarding the safety and efficacy of medicines in centenarians and near centenarians is limited. One study found that out of more than 50,000 randomised controlled trials published between 1990 and 2002, only 84 focussed on people aged 80 years and over, and the mean age of patients included in these studies was 83 years [34]. Safety of drug therapy in centenarians and near centenarians is of particular concern due to the increased risk of adverse drug reactions associated with increasing age [35]. Efficacy of drug therapy in centenarians and near centenarians is also an important consideration. It has been suggested that GPs may be less willing to prescribe medicines for the very old in the absence of efficacy data [36], particularly given the increased risk of adverse effects in this population[37]. Results of our study have highlighted that nearly all centenarians and near centenarians use medicines so it is important that future research focuses on the safety and efficacy of medicines in this age group.

Our study is limited by lack of information on the actual health conditions and comorbidities suffered by centenarians and near centenarians. It is possible that low use of some health resources in our study reflects the inability to access required services. It has been suggested that lower rates of hospitalisation amongst very old people may not reflect better health, but rather the preference of families and doctors to care for the very old in the home [26,38]. We were unable to determine whether this influenced results of our study.

Although we reported the mean number of unique medicines dispensed to centenarians and near centenarians, and the proportion dispensed each class of medicine; we did not describe persistence with drug therapy or differences in doses used by centenarians and near centenarians in comparison to other elderly people. This limitation has also been evident in prior studies of centenarians, which have described the proportion of centenarians using medicine by drug class, but not dosage or duration of therapy [3,9,10]. This is an important consideration and should be the focus of future research, because safe doses of medicine may be lower and appropriate duration of therapy may differ between centenarians, near centenarians and younger patients.

Although our cohort was limited to veterans, results are likely to be applicable to other elderly Australians. Age specific comparisons of DVA gold card holders with the wider Australian population have shown that DVA gold card holders with no service related disability have a similar number of GP visits (rate ratio 0.99, p > 0.5) and slightly fewer hospitalisations (rate ratio 0.97, p < 0.05) per year compared to other elderly Australians [39]. The likelihood of receiving a prescription at a GP visit is similar for the DVA population and the wider Australian population [39]. Our results are likely to be applicable to other Australian centenarians and near centenarians.

## Conclusion

Our study characterised health service and medicine use amongst Australian veteran centenarians and near centenarians. Compared to those aged 65 to 74, a similar or lower proportion of centenarians and near centenarians accessed the health services studied, and those who accessed services did so a similar number of times in comparison to the younger age group. Our results support the findings of other studies which suggest that longevity is not necessarily associated with excessive health service use.

## Competing interests

The authors declare that they have no competing interests.

## Authors' contributions

ER conceived of and designed the study, analysed the data and helped to draft the manuscript. LK drafted the manuscript and participated in data analysis. ER performed the statistical analyses. PR and AG critically revised the manuscript for important intellectual content and assisted in study design. All authors read and approved the final manuscript.

## Pre-publication history

The pre-publication history for this paper can be accessed here:

http://www.biomedcentral.com/1471-2318/10/83/prepub
